# Evidence from a Randomized Trial That Simvastatin, but Not Ezetimibe, Upregulates Circulating PCSK9 Levels

**DOI:** 10.1371/journal.pone.0060095

**Published:** 2013-03-27

**Authors:** Heiner K. Berthold, Nabil G. Seidah, Suzanne Benjannet, Ioanna Gouni-Berthold

**Affiliations:** 1 Charité University Medicine Berlin, Evangelical Geriatrics Center Berlin (EGZB), Berlin, Germany; 2 Charité University Medicine Berlin, Virchow Clinic Campus, Lipid Clinic at the Interdisciplinary Metabolism Center, Berlin, Germany; 3 Laboratory of Biochemical Neuroendocrinology, Clinical Research Institute of Montreal, Montreal, Quebec, Canada; 4 University of Cologne, Center for Endocrinology, Diabetes and Preventive Medicine, Cologne, Germany; Nihon University School of Medicine, Japan

## Abstract

**Background:**

Proprotein convertase subtilisin/kexin type 9 (PCSK9) is a secreted inhibitor of the low-density lipoprotein (LDL) receptor and an important regulator of LDL metabolism. Elevated PCSK9 levels have been associated with cardiovascular risk. The purpose of this study was to investigate how ezetimibe and simvastatin, alone and in combination, affect PCSK9 circulating concentrations.

**Methods:**

A single center, randomized, open-label parallel 3-group study in healthy men (mean age 32±9 years, body mass index 25.7±3.2 kg/m^2^) was performed. Each group of 24 subjects was treated for 14 days with either simvastatin 40 mg/d, ezetimibe 10 mg/d, or with both drugs. Multivariate analysis was used to investigate parameters influencing the change in PCSK9 concentrations under treatment.

**Results:**

The baseline plasma PCSK9 concentrations in the total cohort were 52±20 ng/mL with no statistically significant differences between the groups. They were increased by 68±85% by simvastatin (*P* = 0.0014), by 10±38% by ezetimibe (*P* = 0.51) and by 67±91% by simvastatin plus ezetimibe (*P* = 0.0013). The increase in PCSK9 was inversely correlated with baseline PCSK9 concentrations (Spearman’s *R* = –0.47, *P*<0.0001) and with the percent change in LDL cholesterol concentrations (Spearman’s *R* = –0.30, *P*<0.01). In multivariate analyses, only baseline PCSK9 concentrations (*β* = –1.68, *t* = –4.04, *P*<0.0001), percent change in LDL cholesterol from baseline (*β* = 1.94, *t* = 2.52, *P* = 0.014), and treatment with simvastatin (*P* = 0.016), but not ezetimibe (*P* = 0.42), significantly influenced changes in PCSK9 levels. Parameters without effect on PCSK9 concentration changes were age, body mass index, body composition, thyroid function, kidney function, glucose metabolism parameters, adipokines, markers of cholesterol synthesis and absorption, and molecular markers of cholesterol metabolism.

**Conclusions:**

Ezetimibe does not increase circulating PCSK9 concentrations while simvastatin does. When added to simvastatin, ezetimibe does not cause an incremental increase in PCSK9 concentrations. Changes in PCSK9 concentrations are tightly regulated and mainly influenced by baseline PCSK9 levels and changes in LDL cholesterol.

**Trial Registration:**

ClinicalTrials.gov NCT00317993

## Introduction

Both, statins, which are cholesterol synthesis inhibitors, and ezetimibe, a cholesterol absorption inhibitor, lower low-density lipoprotein (LDL) cholesterol by 40–60% and 20%, respectively. These drugs are often administered together in order to achieve a further decrease in LDL cholesterol levels, when clinically necessary [1].

Proprotein convertase subtilisin/kexin type 9 (PCSK9) is a secreted protein produced mainly in the liver, which binds to the hepatic LDL receptor (LDLR) and targets it for degradation [2]. Gain-of-function mutations of PCSK9 are associated with familial hypercholesterolemia and premature cardiovascular disease [3], while PCSK9 deficiency leads to low LDL cholesterol concentrations and protection against cardiovascular disease [4]. PCSK9 concentrations have been associated with response to statins [5] and with major cardiovascular events [6]. Statins have been shown to upregulate both LDLR and PCSK9 [7]. In turn, increases in PCSK9 concentrations may limit the beneficial effects of statins [8], [9], although this observation is not supported by all studies [10].

These data indicate that the function of circulating PCSK9 is physiologically and clinically significant. Therefore, it would be of interest to investigate how lipid-modifying pharmacological agents affect PCSK9 concentrations. While statins have been shown to increase PCSK9 [10], there are very few data regarding the effects of ezetimibe (alone or combined with a statin) on PCSK9 concentrations [11]. Moreover, it is unknown what other parameters influence changes in PCSK9 concentrations under lipid-lowering therapy. The present randomized study examined the effect of ezetimibe, alone or in combination with simvastatin, on circulating PCSK9 levels.

## Methods

### Study Design and Subjects

The study design has been published before [12], [13]. The protocol for this trial and supporting CONSORT checklist are available as supporting information; see Checklist S1 and Protocol S1. In brief, 72 healthy male subjects were randomized to 3 treatment groups to receive in an open-label design for 14 days at an allocation ratio of 1∶1:1 either ezetimibe 10 mg/d, simvastatin 40 mg/d, or both drugs. Randomization was performed according to a predetermined random list (balanced 6-block design) by use of sealed envelopes. Inclusion criteria were age between 18 and 60 years, body mass index (BMI) between 18.5 and 30 kg/m^2^, fasting LDL cholesterol concentrations <190 mg/dL, fasting triglycerides <250 mg/dL and normal blood pressure (<140/90 mmHg). Excluded from the study were subjects who had received lipid-lowering drugs within 12 weeks prior to study entry, those with a history of excessive alcohol intake, liver disease, renal dysfunction (glomerular filtration rate <60 mL/min), rheumatologic disease, coronary heart disease, diabetes or other endocrine disorders, eating disorders, history of recent substantial (>10%) weight change, history of obesity (BMI>35 kg/m^2^) or taking medications known to affect lipoprotein metabolism, glucose metabolism, or the immune system. All patients were advised to keep their usual dietary habits throughout the trial. Blood was drawn in the morning after a 12-h fast at days 1 (before the initiation of treatment) and 15 (at the end of the 2-week treatment period). The original trial has been registered at ClinicalTrials.gov NCT00317993. The study was performed at the outpatient clinic of the University of Cologne in 2005 and the protocol was approved by the Ethics Committee of the University of Cologne, and all subjects gave written informed consent. The sponsors had no influence on study design, analyses or interpretation of the data.

### Analytical Measurements

Human PCSK9 concentrations were measured as previously described [14]. The polyclonal antibody used was prepared in rabbit and directed against affinity purified proPCSK9 (aa 31–454) produced in bacteria [15]. The antibody recognizes both the prosegment (aa 31–152) and the catalytic subunit (aa 153–454) of PCSK9 and not its C-terminal CHRD (aa 455–672). In short, LumiNunc Maxisorp white assay plates (Nunc, Denmark) were coated with 0.5 µg per well of our human PCSK9 antibody by incubation at 37°C for 3 h in PBS (10 mM NaPO_4_, 0.15 M NaCl, pH 7.4) containing sodium azide (1 g/L), then stored at 4°C. The plates were washed six times before use with PBS-containing Tween 20 (0.5 mL/L) and then incubated for 1 h at room temperature with blocking buffer (PBS, casein 0.1%, merthiolate 0.01%). Calibrators were prepared using serial dilutions of recombinant PCSK9 in dilution buffer (PBS, urea 1.8 M, 0.25% BSA, 0.5 ml/L Tween 20, and 0.01% merthiolate). Plasma samples were diluted 1∶20 in dilution buffer without BSA. Calibrators and plasma samples were incubated for 30 min in a water bath at 46°C prior to plate addition (100 µL) in duplicate. We found that pre-incubation at 46°C enhances the antigen recognition by the antibody. The plates were incubated overnight at 37°C with shaking. After washing, 100 µL of human PCSK9-Ab-HRP diluted 1∶750 was added and incubation continued for 3 h at 37°C with shaking. Finally, after washing, 100 µL of substrate (SuperSignal™ ELISA Femto Substrate, Pierce) was applied to each well. Chemiluminescence was quantitated on a Pherastar luminometer (BMG Labtech). A standard curve was established using a conditioned medium containing recombinant human PCSK9 as described previously [14], where the linear portion of the assay occurs between 2.5–20.0 ng/mL of human PCSK9.

Analytical details for the determination of non-cholesterol sterols (as markers of cholesterol synthesis and absorption) and quantitative real-time PCR of mRNAs of HMG-CoA reductase, LDL receptor, Niemann-Pick C1-like protein 1 (NPC1L1), and PCSK9 from plasma mononuclear cells as well as flow-cytometry for cell surface LDLR protein expression have been described in detail previously [1]. All other biochemical analyses were made in the core laboratory of the Cologne University Medical Center using standard laboratory procedures [16].

### Statistical Analyses

Statistical analyses were performed using Stata 12 (StataCorp LP, College Station, TX). Descriptive data are given as means ± SD or proportions (in percent), unless otherwise indicated. The primary outcome parameter of the parent trial was change in LDL cholesterol. The primary outcome parameter of the current post-hoc analysis is change in PCSK9 concentrations. Thus, no sample size calculation was performed for this latter outcome. Associations between baseline PCSK9 concentrations and baseline clinical and biochemical parameters were examined using correlation analyses (Spearman’s rank test). Bivariate regression models were performed to investigate which parameters (baseline and on-treatment values) influence the change of PCSK9 concentrations from baseline. In a final model, multivariate regression analyses were performed. In these analyses, also the baseline values and the effects of the 2 treatments, ezetimibe and simvastatin, were modeled. Statistical significance was assumed at *P*<0.05 using 2-sided tests.

## Results

**Figure 1:**
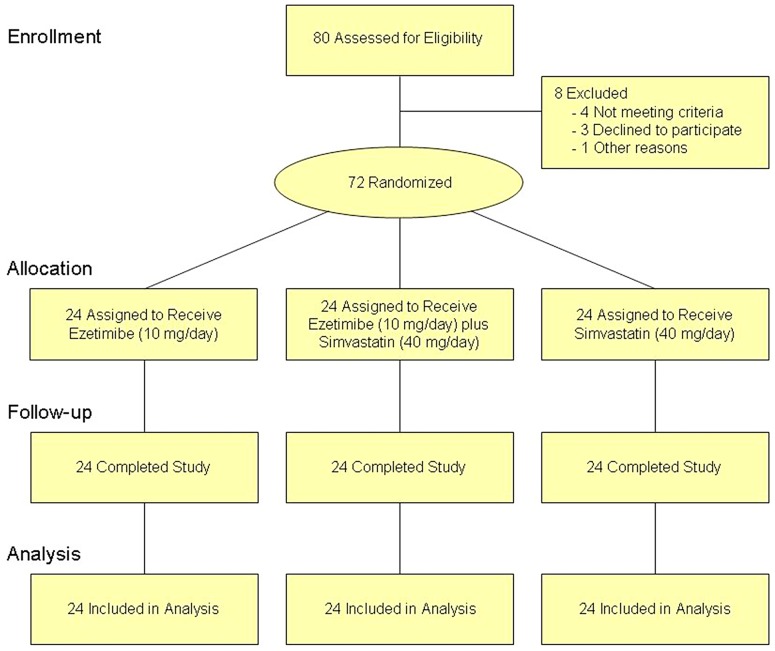
CONSORT flow diagram: Flow of participants through the trial.

**Figure 2:**
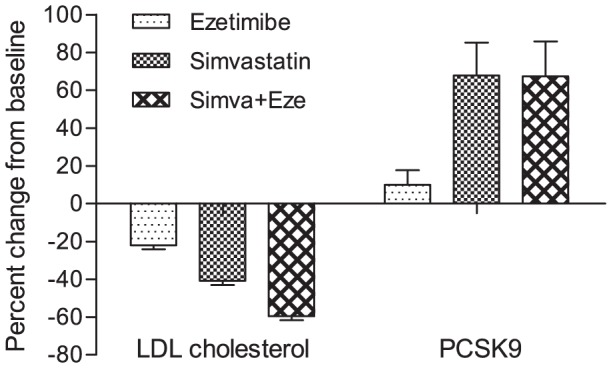
Change in LDL cholesterol and PCSK9 from baseline according to treatment groups. Data are means ± SEM. The decrease in LDL cholesterol was significant in all groups (all *P*<0.0001), the increase in PCSK9 was significant in the simvastatin and in the combination groups (*P*<0.005), but not in the ezetimibe group.

Figure 1 shows the flow of participants through the trial. Within the total cohort, PCSK9 baseline concentrations were 51.7±19.9 ng/mL with no statistically significant difference between the 3 groups. Table 1 summarizes the baseline clinical and biochemical data in the three groups. There were no important differences between the groups. Table 2 shows the results of the correlation analyses between baseline parameters and PCSK9 concentrations. Mean baseline LDL cholesterol concentrations were 111±30 mg/dL with no significant difference between the 3 groups. As expected, LDL cholesterol decreased by 22, 41 and 60% in the ezetimibe, simvastatin and ezetimibe plus simvastatin groups, respectively (Figure 2). The changes in PCSK9 concentrations were +9.9±38% (n.s.), +67.8±85.2% (*P* = 0.0012) and +67.3±90.7% (*P* = 0.0013) in the 3 groups, respectively (Figure 2).

**Table 1:** Baseline data in the three treatment groups.

Parameter	Ezetimibe 10 mg/day	Simvastatin 40 mg/day	Ezetimibe 10 mg/dayplus simvastatin40 mg/day	*P* value*
*Anthropometric and clinical parameters*				
Age (years)	28.6±6.6	31.9±8.8	34.1±11.2	0.11
Body mass index (kg/m^2^)	25.0±3.3	26.4±3.2	25.8±3.1	0.35
Body fat (%)	20.6±5.4	22.5±5.7	21.1±6.2	0.48
Estimated glomerular filtration rate (mL/min)	132±19	143±26	129±26	0.11
Thyroid stimulating hormone (mU/L)	1.59±0.79	1.54±0.66	1.83±0.99	0.42
High-sensitivity CRP (mg/L)	0.63±0.74	1.10±1.20	0.53±0.69	0.072
Plasma PCSK9 (ng/mL)	51.0±19.9	47.2±18.2	56.9±21.0	0.24
*Lipoproteins*				
Total cholesterol (mg/dL)	180±28	194±34	194±41	0.30
LDL cholesterol (mg/dL)	105±23	113±30	116±35	0.40
HDL cholesterol (mg/dL)	64±13	65±18	61±14	0.66
Triglycerides (mg/dL)	78±32	101±45	106±48	0.051
*Glucose metabolism and adipokines*				
Fasting glucose (mg/dL)	87±6	86±7	89±9	0.34
Fasting insulin (mU/L)	11.3±13.6	8.7±2.5	9.7±5.1	0.56
HOMA index†	2.5±2.8	1.9±0.7	2.1±1.1	0.57
Leptin (µg/mL)	2.6±2.9	3.4±3.0	2.8±2.5	0.63
High-molecular weight adiponectin (µg/mL)	2.8±2.3	2.8±2.1	2.5±1.6	0.86
*Non-cholesterol sterols*				
Lathosterol**	127±37	156±45	130±33	0.022
Desmosterol**	67±13	74±15	78±31	0.19
Cholestenol**	11±4	15±5	12±4	0.008
Cholestanol**	150±32	140±26	145±26	0.49
Sitosterol**	125±42	131±53	123±34	0.79
Campesterol**	206±77	211±99	208±73	0.98
Campesterol/lathosterol (mg/mg)	1.81±0.99	1.53±1.06	1.91±1.82	0.59
*Molecular markers of cholesterol metabolism*				
LDL receptor protein (PBMC)‡	17.9±8.6	16.5±7.6	18.4±11.8	0.81
HMG-CoA reductase mRNA¶	3.35±1.07	3.21±1.53	3.28±1.44	0.94
LDL receptor mRNA¶	0.59±0.44	0.45±0.25	0.52±0.23	0.33
NPC1L1 mRNA¶	25±19	45±58	28±20	0.17
PCSK9 mRNA¶	1.71±0.15	1.68±0.09	1.69±0.10	0.59

Values are means ± SDs.

* ANOVA *P* value.

** The data indicate the ratio of the respective non-cholesterol sterol to cholesterol (µg/mg) ×100.

† Homeostasis model assessment.

‡ LDL receptor protein is given as flow cytometry-specific fluorescence, calculated by subtracting the nonspecific fluorescence intensity from the total fluorescence intensity.

¶ Gene expression is given as number of the respective mRNA copies divided by the number of copies of the TATA housekeeping gene mRNA.

**Table 2:** Baseline mean values and correlation analyses between baseline values and baseline PCSK9 concentrations.

Parameter	Mean ± SD	Spearman’s *rho*	*P* value
*Anthropometric and clinical parameters*			
Age (years)	31.5±9.2	0.19	0.12
Body mass index (kg/m^2^)	25.7±3.2	0.06	0.61
Body fat (%)	21.3±5.6	0.03	0.80
Estimated glomerular filtration rate (mL/min)	135±25	0.06	0.49
Thyroid stimulating hormone (mU/L)	1.65±0.82	–0.11	0.34
High-sensitivity CRP (mg/L)	0.75±0.93	–0.01	0.92
*Lipoproteins*			
Total cholesterol (mg/dL)	189±35	0.13	0.28
LDL cholesterol (mg/dL)	111±30	0.10	0.39
HDL cholesterol (mg/dL)	64±15	0.30	0.01*
Triglycerides (mg/dL)	95±43	–0.16	0.18
*Glucose metabolism and adipokines*			
Fasting glucose (mg/dL)	88±8	0.05	0.70
Fasting insulin (mU/L)	9.9±8.4	0.04	0.71
HOMA index†	2.2±1.8	0.07	0.57
Leptin (µg/mL)	3.0±2.8	0.08	0.52
High-molecular weight adiponectin (µg/mL)	2.7±2.0	0.18	0.13
*Non-cholesterol sterols*			
Lathosterol**	138±40	–0.24	0.04*
Desmosterol**	71±14	–0.20	0.09*
Cholestenol**	12.8±4.5	–0.25	0.03*
Cholestanol**	145±28	0.13	0.26
Sitosterol**	126±43	0.10	0.39
Campesterol**	208±83	0.13	0.29
Campesterol/lathosterol (mg/mg)	1.75±1.33	0.21	0.08*
*Molecular markers of cholesterol metabolism*			
LDL receptor protein (PBMC)‡	17.6±9.3	–0.26	0.04*
HMG-CoA reductase mRNA¶	3.3±1.3	0.17	0.17
LDL receptor mRNA¶	0.52±0.32	–0.03	0.78
NPC1L1 mRNA¶	33±31	–0.21	0.09*
PCSK9 mRNA¶	1.7±0.1	0.06	0.60

Values are means ± SDs, Spearman’s correlation coefficients *rho* between baseline plasma PCSK9 concentrations and clinical or biochemical parameters, and the associated *P* values. *indicates *P* values <0.10.

** The data indicate the ratio of the respective non-cholesterol sterol to cholesterol (µg/mg) ×100.

† Homeostasis model assessment.

‡ LDL receptor protein is given as flow cytometry-specific fluorescence, calculated by subtracting the nonspecific fluorescence intensity from the total fluorescence intensity.

¶ Gene expression is given as number of the respective mRNA copies divided by the number of copies of the TATA housekeeping gene mRNA.

Baseline PCSK9 levels were not influenced by age, body mass index, percent body fat, estimated glomerular filtration rate, thyroid-stimulating hormone, or high-sensitivity CRP (Table 2). There was a significant positive correlation with HDL cholesterol and weak but non-significant positive correlations with total and LDL cholesterol, with parameters of glucose metabolism (fasting glucose and insulin, HOMA index) and with adipokines (leptin, high-molecular weight adiponectin). The correlations with markers of endogenous cholesterol synthesis (lathosterol, desmosterol and cholestenol) were negative and significant, while correlations with markers of cholesterol absorption (cholestanol, sitosterol and campesterol) were slightly positive but non-significant. There was a significant positive correlation with the overall ratio of campesterol to lathosterol. A high c/l ratio was shown previously to indicate a high rate of intestinal absorption of cholesterol, whereas a low ratio indicates a low absorption [17]. There was a significant negative correlation with LDLR protein expression.

**Figure 3:**
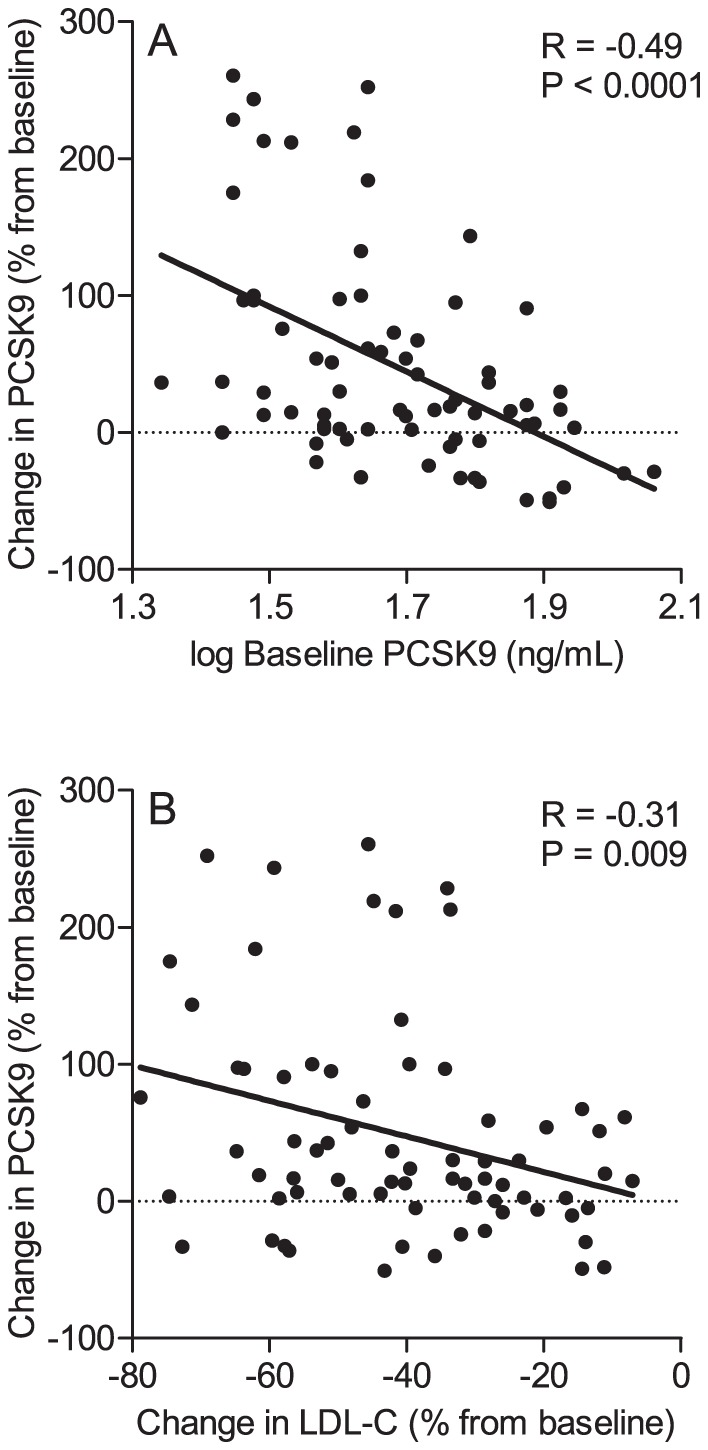
Change in PCSK9 from baseline according to baseline PCSK9 and baseline LDL cholesterol. (A) Correlation between baseline PCSK9 concentrations (log-transformed) and percent change in PCSK9 from baseline in the total cohort (N = 72). (B) Correlation between percent change in LDL cholesterol and percent change in PCSK9 from baseline in the total cohort (N = 72). The *R* values shown are the ones from linear regression analyses. The corresponding *R* values from Spearman’s rank correlation analyses are –0.47 (*P*<0.0001) or –0.30 (*P*<0.001), respectively.

The increase in PCSK9 was strongly inversely correlated with baseline PCSK9 (Spearman’s *rho* = –0.47, *P*<0.0001) and with the percent change in LDL cholesterol (Spearman’s *rho* = –0.30, *P*<0.01), (Figure 3). The effects of individual parameters shown in Table 2 on the percent change in PCSK9 levels were further investigated in multiple regression analyses. All analyses were performed using baseline and change of the respective parameter during treatment. Adjustments were made for baseline PCSK9 concentrations and drug treatment. In the final model, only baseline PCSK9 concentrations (*β* = –1.68, *t* = –4.04, *P*<0.0001) and the percent change in LDL cholesterol from baseline (*β* = 1.94, *t* = 2.52, *P* = 0.014) had a significant influence on change in PCSK9 concentrations. Moreover, simvastatin (*P* = 0.016), but not ezetimibe (*P* = 0.42), had a statistically significant effect. The parameters of the final model explained a substantial and significant proportion of the variance in change of PCSK9 concentrations (*R* ^2^ = 0.31, *F*(4,67) = 7.62, *P*<0.00001).

**Figure 4:**
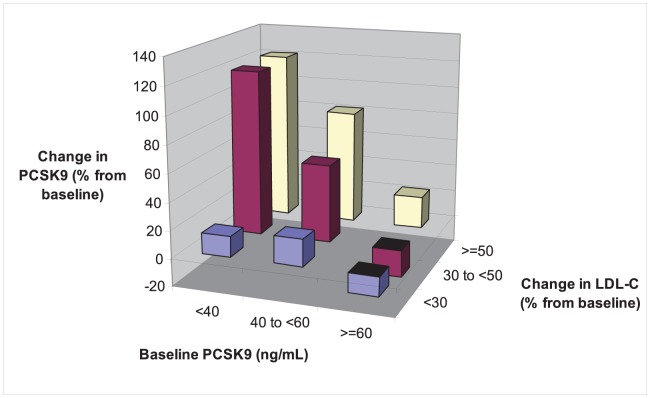
Change in PCSK9 concentrations (in percent from baseline) according to baseline PCSK9 concentrations and change in LDL cholesterol (in percent from baseline). Subjects were divided approximately in tertiles: baseline PCSK9<40 ng/mL, 40 to <60 ng/mL, or ≥60 ng/mL; change in LDL cholesterol from baseline <30%, 30 to <50%, or ≥50%.

As shown in Figure 4, the strongest increase in plasma PCSK9 was observed in subjects with low baseline PCSK9 (<40 ng/mL) and a pronounced LDL cholesterol-lowering effect under treatment (≥50% from baseline). In subjects with high baseline PCSK9 (≥60 ng/mL), PCSK9 concentrations were hardly affected (–20 to +23%), even in the presence of pronounced LDL-lowering. *Vice versa*, when PCSK9 was low at baseline (<40 ng/mL), even a moderate LDL cholesterol decrease (30 to 50% from baseline) led to robust upregulation in PCSK9 concentrations (up to 120%). Multivariate analyses indicated that significant changes in PCSK9 by lipid-lowering medication were seen *only* in subjects receiving simvastatin (either as monotherapy or in combination), but *not* in subjects receiving ezetimibe.

**Figure 5:**
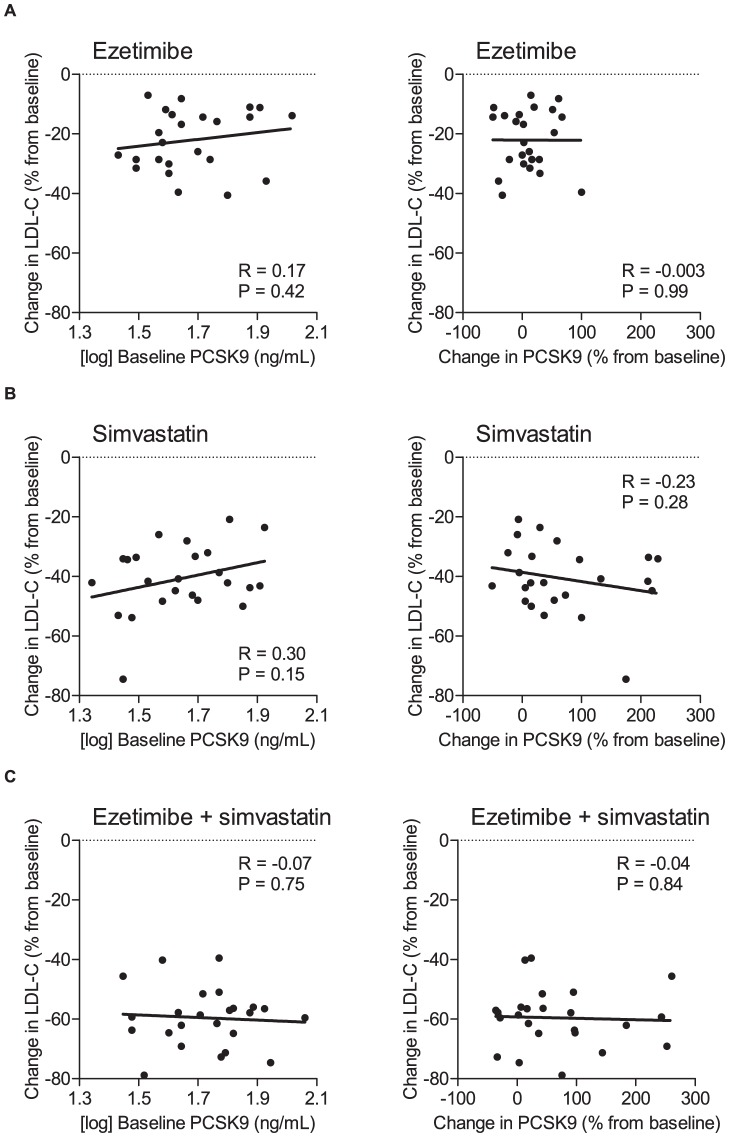
Correlation between baseline PCSK9 concentrations (left panels) and percent change in PCSK9 from baseline (right panels) and percent change in LDL cholesterol from baseline. (A) ezetimibe, (B) simvastatin, (C) ezetimibe plus simvastatin.

Further, to test the hypothesis that individuals with higher baseline levels of PCSK9 respond less well to simvastatin [18] and *vice versa* [19], we examined the relationship between PCSK9 levels and responses to ezetimibe, simvastatin and ezetimibe plus simvastatin (Figure 5). No significant correlations were observed between baseline PCSK9 levels and the response to LDL-lowering treatment.

## Discussion

The current randomized trial investigated the effects of the 2 lipid-lowering drugs simvastatin and ezetimibe, alone and in combination, on PCSK9 concentrations. A multitude of clinical, biochemical and molecular parameters were assessed as covariates. The average baseline PCSK9 concentrations were very similar to the levels observed by others using the same method of PCSK9 measurement [10], [14]. The main finding of this study is that the change in PCSK9 concentrations induced by lipid-lowering is influenced mostly by baseline PCSK9 and by the decrease in LDL cholesterol. Other clinical and biochemical parameters had no effect. Of the 2 drugs tested, only simvastatin had significant effects on PCSK9 levels. Interestingly, the increase in PCSK9 levels observed with the standard dose of simvastatin, 40 mg/d, was not further enhanced by the addition of ezetimibe despite its incremental effect on LDL cholesterol lowering. Moreover, ezetimibe monotherapy had no significant effect on PCSK9 concentrations, in concordance to recent findings of Lakoski *et al.* [20], although it lowered LDL cholesterol levels by 20%.

A possible explanation for these findings is that, since statins upregulate PCSK9 expression, treatment with 40 mg simvastatin daily for 2 weeks is sufficient to maximally increase circulating PCSK9 concentrations to a plateau, with no further increase possible by further LDL cholesterol lowering. Huigen *et al.* also observed this plateau effect when comparing atorvastatin 10 mg and 80 mg [6]. On the other hand, a low dose of a statin, simvastatin 10 mg daily, has been found insufficient to increase PCSK9 levels [20]. Thus, PCSK9 seems to be tightly regulated within a certain range of statin-induced LDL cholesterol decrease. Ezetimibe may not increase PCSK9 concentrations because of its weak LDL-lowering effects, which seemingly are not strong enough to upregulate PCSK9 expression. Alternatively, a reason may be the absence of pleiotropic effects in comparison to statins. Statins may stimulate PCSK9 expression independently of lipid-lowering –*e.g.*, they upregulate PPAR-α/β/γ/δ, which are involved in the regulation of PCSK9 expression in the liver [21], [22]. The latter argument may also explain why further LDL-lowering by ezetimibe when added to a statin does not result in further PCSK9 increase. The correlation we observed between change in plasma PCSK9 levels and the percent reduction of LDL cholesterol from baseline is in accordance to recent findings by others [14], [23]. We surmise that this correlation is driven by parallel upregulation of PCSK9 and LDLR mRNAs in response to the intracellular LDL cholesterol-lowering effect of statins.

Our study has limitations. Firstly, no *a priori* power calculations were made for changes in PCSK9 concentrations because the primary outcome parameter of the parent trial was change in LDL cholesterol. Secondly, treatment duration was relatively short. However, longer treatment periods with ezetimibe have shown similar results [20] and the maximal LDL cholesterol-lowering effect of statins and ezetimibe is achieved within 2 weeks [24], [25]. Furthermore, due to the relatively small size of our study existing associations may have been underestimated or missed. Our findings need to be confirmed in larger trials. The open-label design of the parent study may have introduced bias. Finally, PBMC might not accurately reflect hepatic PCSK9 gene and protein expression under all circumstances or with all forms of pharmacological intervention. However, recent evidence strongly supports the use of PBMC for the study of genes related to hepatic cholesterol metabolism [26], [27] and PBMC have been used for this purpose in many studies [1], [28]–[30]. Moreover their use has been advocated as a convenient means to provide organ specific data without organ tissue itself [31], [32].

Strengths of the study include its randomized design, robust statistical methodology, blinded measurements of plasma PCSK9 concentrations, and the use of a ‘drug-naive’ population, devoid of co-medications and co-morbidities, which could potentially alter lipid metabolism, and excellent treatment adherence (pill count 99.1%). Moreover, this is the first randomized trial examining multiple clinical and biochemical parameters possibly modulating PCSK9 concentrations, ranging from gene expression to markers of cholesterol absorption and synthesis, to adipokines, glucose metabolism and other parameters, in one cohort.

### Conclusions

In conclusion, the current data support and expand previous reports suggesting that ezetimibe, alone or combined with simvastatin, is not associated with an increase in PCSK9. These findings may help identify those individuals that would benefit most from treatment with PCSK9 antibodies, which are in clinical development. Finally, our results indicate that changes in PCSK9 concentrations during lipid-lowering treatment are tightly regulated and are mainly influenced by baseline PCSK9 levels and statin-induced changes in LDL cholesterol, underlining the relevance of genetic variations in PCSK9.

## Supporting Information

Checklist S1 CONSORT Checklist.
(DOC)

Protocol S1 Trial Protocol.
(PDF)
