# Supplementary material for: Evidence from a Randomized Trial That Simvastatin, but Not Ezetimibe, Upregulates Circulating PCSK9 Levels
Source: PLoS One. 2013 Mar 27;8(3):e60095. doi: 10.1371/journal.pone.0060095 (PMC3609731; doi:10.1371/journal.pone.0060095)
Supplement: Protocol S1 — Trial Protocol. (PDF) [file pone.0060095.s002.pdf]

Application to the Ethics Committee of the Medical Faculty of the University of Cologne

Chairman: Prof. Dr. med. M. Staak

A. Formal

1. Title of the study

Effects of ezetimibe and simvastatin on LDL receptor protein expression and on LDL receptor and HMG-CoA reductase mRNA expression in mononuclear cells: a randomized controlled study in healthy men

2. Applicant and responsible primary medical investigator (Leiter der klinischen Prüfung im Sinne des AMG), deputy and further study physicians

Priv.-Doz. Dr. Ioanna Berthold
Fachärztin für Innere Medizin/Endokrinologie
Medizinische Klinik II und Poliklinik für Innere Medizin
Direktor: Prof. Dr. med. W. Krone
Universität zu Köln
Joseph-Stelzmann-Straße 9
50924 Köln
Tel.: 0221/478-4088
Fax: 0221/478-6458
E-mail: ioanna.berthold@uni-koeln.de

Deputy:

Prof. Dr. med. Wilhelm Krone

Further study physicians:

Prof. Dr. med. Heiner K. Berthold
Arzneimittelkommission der deutschen Ärzteschaft (Köln)
Scientific consulting, study performance, biometry

Prof. Dr. med. Yon Ko
Medizinische Fakultät der Universität Bonn
Laboratory analyses

3. Art und Zahl der Prüfstellen

1 Center (monocenter study)

4. Has an application of similar content been filed before with another ethics committee?

No.

5. Written consent of the director of the respective clinic

has been filed (Appendix 1)

6. and 7. Documentation of study-related additional costs, financing plan, third money

The trial will be sponsored by MSD Sharp & Dohme GmbH, München. For accounting of the study an account has been established with the administration of the University clinic Cologne (account no. 3620/1394/31 (letter of the administrative director of 10 December 2003)

8. Consulting fee

Written agreement that in case of an industry-sponsored study the consulting fee will be booked directly from an account of the administration of the University clinic Cologne (Mrs. Landvogt) (Appendix 2).

10. Multicenter trials

This trial is a monocenter study.
Approximately 60 subjects will be recruited at the University of Cologne.

11. Statement

Herewith I declare before the ethics committee of the medical faculty of the University of Cologne that I will garantuee the technical, staff and organizational requirements of the clinical trial.

Cologne, 09 January 2004

Priv.-Doz. Dr. Ioanna Berthold
Internist/Endocrinologist

B. Description of the trial

1. Scientific goals

see Appendix 3

2. Study protocol

see Appendix 4

3. Planned invasive measures and burden expected for the participants

The subjects will take oral study medication through the treatment period of 2 weeks (1 or 2 tablets daily). Furthermore, blood will be drawn three times, twice at baseline and once at the end of the study.

4. Patient selection

see Appendix 5

5. Kind of study (in drug studies: phase?)

a) diagnostic study?	no
b) therapeutic study	no
c) tolerability study?	no
d) epidemiological study?	no
e) other study?	yes

This is a pharmacodynamic pilot study with 2 approved drugs (phase 4) which should extend current knowledge about effects and mechanisms of action. The study is an investigator-initiated trial.

6. Which laws and regulations apply?

a) Arzneimittelgesetz?	yes
b) Strahlenschutzgesetz?	no
c) Röntgenverordnung?	no
d) Medizingeräte-Verordnung?	no
e) Grundsätze	yes
f) Meldung RP	yes

7. Which prestudies have been done?

Two approved drugs will be investigated which are widely marketed. There were no specific prestudies.

Pharmacologic-toxicologic studies:

a) performed?
Yes, drug approval files are available at the Federal Institute for Drugs and Medical Devices (BfArM). There is an abundance of medical scientific literature on ezetimibe and simvastatin.

b) Results filed with the German drug agency?
Yes, within the framework of drug approval (Ezetrol®, Zocor®)

c) Summary of relevant results for the performance of this study
see general scientific literature on ezetimibe/simvastatin

8. Possible complications and/or risks?

Two approved and marketed lipid-lowering drugs will be used, whose safety and tolerability has been documented in the international literature and in the approval files. For further information see Fachinformationen (Appendix 6).

9. Risk-benefit-considerations

This is a study in healthy subjects which is intended to increase biochemical insights. The subjects will have no direct benefit from participating in the trial but the burden of short-term medication will be justifiable in view of the expected scientific results.

10. Interim analysis and criteria for early termination

There will be no interim analysis. The planned number of participants and the analyses will have to be reached before the study will be evaluated.

The termination criteria for the individual participant will be according to general clinical practice using ezetimibe or simvastatin.

Early termination of the whole trial will not be expected, therefore no specific criteria will be defined for this event. The decision about an early termination will be made by the principle investigator.

11. and 12. Informed consent

see Appendix 7

13. Professional secrecy

see Appendix 7

14. Insurance?

see Appendix 8

15. Financial plan?

Financing the study is garantueed (see A.6 and A.7).

C. Kurzfassung (German)

Ezetimib senkt das Gesamtcholesterin und das LDL-Cholesterin über eine Hemmung der intestinalen Cholesterinresorption, wodurch es zu einem kompensatorischen Anstieg der endogenen Cholesterinsynthese kommt. Die genauen zugrunde liegenden regulatorischen Mechanismen des durch Ezetimib induzierten Anstiegs der Cholesterinsynthese und der Abnahme des LDL-Cholesterin sind nicht bekannt. Darüber hinaus wurde nie untersucht, ob Veränderungen der Expression des LDL-Rezeptors zum Cholesterin senkenden Effekt von Ezetimib beitragen.

Die vorliegende Studie untersucht, ob Änderungen in der LDL-Rezeptor-Expression und in den Konzentrationen der HMG-CoA-Reduktase-mRNA unter Behandlung mit Ezetimib zu beobachten sind. Zum Vergleich werden die Wirkungen einer Behandlung mit Simvastatin und die kombinierte Gabe von Simvastatin und Ezetimib untersucht. Da das Profil der mRNA-Expressionen Informationen über die Wirkungen auf der Transkriptionsebene, aber nicht notwendigerweise auch auf der translationalen Ebene gibt, werden auch Veränderungen des LDL-Rezeptor-Proteins auf der Zelloberfläche von mononukleären Zellen untersucht. Als ein funktioneller Marker der HMG-CoA-Reduktase-Aktivität wird die Ratio der Konzentration von Lathosterin zu Cholesterin im Serum verwendet, da diese mit der Aktivität der HMG-CoA-Reduktase korreliert und auch als Marker der Gesamtcholesterinsynthese herangezogen werden kann.

In diesem Zusammenhang konnte gezeigt werden, dass pflanzliche Sterole, die auch über eine Hemmung der intestinalen Cholesterinresorption wirken, die Synthese von Gesamtcholesterin und LDL-Cholesterin steigern, die Konzentrationen der LDL-Rezeptor-mRNA steigern und auch die Konzentrationen des LDL-Rezeptor-Proteins steigern, aber keinen signifikanten Effekt auf die Expression der HMG-CoA-Reduktase oder Aktivität in peripheren mononukleären Blutzellen haben.

Das Ziel dieser prospektiven randomisierten Parallelgruppenstudie ist es zu untersuchen, ob die Behandlung mit Ezetimib Veränderungen in der HMG-CoA-Reduktase-Aktivität/-Expression oder in der LDL-Rezeptor-Expression/-Protein-Konzentration in mononukleären Blutzellen hat. Zu diesem Zweck werden drei Parallelgruppen mit je 20 erwachsenen Männern (gesund, ohne Medikation, weitgehend normalgewichtig) gebildet. Eine Gruppe wird mit Ezetimib 10 mg/Tag, die andere mit Simvastatin 40 mg/Tag und die dritte mit Ezetimib plus Simvastatin in den genannten Dosierungen behandelt. Die Behandlungsdauer beträgt 2 Wochen. Vor Beginn der Behandlung sowie am Ende der Behandlung werden jeweils Blutproben entnommen, die bei –80 °C eingefroren werden. Die in dieser Studie bestimmten Parameter sind die Lipoproteinkonzentrationen, die Isolation von mononukleären Zellen, die Messung der LDL-Rezeptor-mRNA in peripheren mononukleären Zellen, die Messung der HMG-CoA-Reduktase-mRNA in peripheren mononukleären Zellen und die Bestimmung der LDL-Rezeptor-Protein-Konzentrationen auf der Oberfläche von peripheren mononukleären Zellen im Blut. Darüber hinaus werden die Konzentrationen von Lathosterin und Cholesterin im Serum bestimmt, deren Ratio als Surrogatmarker für die HMG-CoA-Reduktase-Aktivität verwendet wird.

Zur Teilnahme an der Studie kommen männliche Probanden in Frage, die mental und linguistisch in der Lage sind, die Ziele der Studie zu verstehen und eine genügende Compliance besitzen, das Studienprotokoll einzuhalten, sich im Alter zwischen 18 und 60 Jahren befinden, in einem allgemein guten Gesundheitszustand befinden (festgestellt aufgrund der Anamnese, der körperlichen Untersuchung und der Laboruntersuchung), einen Body mass index zwischen 18,5 und 30 kg/m^2 haben, ein LDL-Cholesterin < 190 mg/dl und Triglyzeride < 250 mg/dl haben, einen normalen Blutdruck (< 140/90 mmHg haben (eine isolierte systolische Hypertonie (RR syst. > 140 mmHg, diast. < 90 mmHg ist akzeptable, falls ansonsten ein ausreichender Gesundheitszustand besteht), und insbesondere keines der folgenden Ausschlusskriterien aufweisen: aktive Lebererkrankung oder ungeklärte und andauernde Erhöhung der Serum-Transaminasen, Cholestase, Myopathie.

Summary

Ezetimibe decreases serum total and LDL cholesterol levels by blocking cholesterol absorption in the intestine, causing a compensatory increase in cholesterol synthesis. The exact underlying regulatory mechanisms of the ezetimibe-induced increase in cholesterol synthesis and decrease in serum LDL cholesterol are not known. In addition, it has never been investigated whether changes in LDL receptor expression contribute to the LDL-lowering effect of ezetimibe, as is the case with other agents causing a decrease in cholesterol absorption such as the plant stanols.

In the present study, we plan to examine changes in LDL receptor and HMG-CoA reductase mRNA concentrations during ezetimibe treatment. For comparison, effects of simvastatin and the combined administration of the two will be investigated. Since mRNA expression profiles provide information about effects at the transcriptional but not necessarily at the translational level, we will also analyze changes in the LDL receptor protein at the cell surface of mononuclear blood cells. As a functional marker for HMG-CoA reductase activity the ratio of serum lathosterol to cholesterol concentration will be used since it correlates with HMG-CoA reductase activity and serves also as a marker of total cholesterol synthesis.

In this regard it has been shown that plant sterols, which also act by blocking intestinal cholesterol absorption, increase cholesterol synthesis, decrease LDL synthesis, increase LDL receptor mRNA levels as well as LDL receptor protein concentrations but have no significant effect on HMG-CoA reductase expression or activity in peripheral blood mononuclear cells. Aim of this prospective randomized parallel study is to examine changes in HMG-CoA reductase activity/expression and in LDL receptor expression/protein concentration in mononuclear blood cells under treatment with ezetimibe.

For this purpose 3 parallel groups of 20 healthy men will be formed. One group will be treated with ezetimibe (10 mg/day), one with 40 mg/day of simvastatin and another with ezetimibe (10 mg/day) plus simvastatin (40 mg/day). Each treatment period will last for 2 weeks. Blood drawing will be performed at baseline (before the initiation of treatment) and at the end of the 2 weeks. (storing of the samples at –80°). The measurements involved in this study include the determination of the lipoprotein concentrations in serum, isolation of the mononuclear cells, measurement of LDL receptor mRNA from the peripheral blood mononuclear cells, measurement of HMG-CoA reductase mRNA levels in peripheral blood mononuclear cells, measurement of the LDL-receptor protein concentrations on the surface of peripheral blood mononuclear cells. Furthermore, the serum latosterol to cholesterol concentrations will be measured as a surrogate marker of the HMG-CoA reductase activity.

Appendix 3:

Scientific Background

Cells in the human body need cholesterol which can be derived from endogenous synthesis or from receptor-mediated uptake of circulating lipoproteins such as intermediate density lipoproteins (IDL) and low density lipoproteins (LDL) (1). Circulating lipoproteins are taken up by various receptors, all with their own specific ligands. LDL, for example, is mainly endocytosed by a specific apoB/E receptor, known as the LDL receptor. Transcription of the LDL receptor gene is up-regulated by low cellular free sterol concentrations and is initiated by activated sterol response element binding proteins (SREBPs) (2). The transcription of the rate-limiting enzyme for endogenous cholesterol synthesis, 3-hydroxy-methyl-glutaryl coenzyme A (HMG-CoA) reductase, is controlled in a similar way (3), although its activity depends on post-transcriptional modifications (4).

The benefits of lipid-lowering therapy on coronary heart disease (CHD) risk have been clearly established in many large-scale primary and secondary prevention trials. Although statins (HMG-CoA reductase inhibitors) have been shown to be effective in lowering LDL cholesterol, many patients do not achieve the standard treatment goals. Although higher doses of statins are more effective in lipid lowering, the risk of serious side effects seems to be dose dependent (5). Therefore the need of additional lipid-lowering compounds, not acting as HMG-CoA reductase inhibitors, has focused attention on drugs with different mechanisms of action such as the inhibition of intestinal cholesterol absorption (6). Intestinal cholesterol absorption shows great inter-individual variation and ranges from 20–80 % (7–9). It is known that a decrease in cholesterol absorption caused by consumption of foods enriched in plant sterols is associated with an increase in cholesterol synthesis (10).

Ezetimibe is a compound of the 2-azetidinone class that has been shown to produce a marked inhibition of intestinal cholesterol absorption of 54 % in humans, a compensatory increase of cholesterol synthesis and a decrease in LDL- and total cholesterol (11). In animal models the ezetimibe-induced inhibition of cholesterol absorption ranges between 92–96 % in a dose range of 1–10 mg/kg (12;13). A dose of 10 mg daily has been shown to decrease LDL cholesterol in the range of 17–20 % (14). It does not affect the absorption of triglycerides or fat-soluble vitamins (11). Studies in bile duct cannulated rats show that intraduodenally delivered ezetimibe undergoes rapid and extensive metabolism to its phenolic glucuronide in the intestine. In the portal vein, more than 95 % of ezetimibe is already glucuronidated and ezetimibe acts directly in the intestine as a glucuronide (15).

The aforementioned observed ezetimibe-induced increase in cholesterol synthesis might explain the favorable effects of co-administration of ezetimibe and statins (16). In this context, in vivo experiments in humans assessing the effect of ezetimibe on the activity of drug-metabolizing enzymes revealed no effect on the activity of the cytochrome P450 (CYP) isoenzymes CYP1A2, CYP2C8/9, CYP2D6, CYP3A4 or N-acetyltransferase (17). Thus, the pharmacokinetic interaction potency of ezetimibe seems to be low. Co-administration of ezetimibe with statins produces an incremental 12–15 % decrease in LDL cholesterol, independent of kind and dose of the co-administered statin (11). When ezetimibe is added to an ongoing statin therapy, LDL cholesterol is incrementally reduced by 21.5 % (relative to on-statin baseline values) compared with placebo (6;11). Thus, because statins can reduce the compensatory increase in the hepatic cholesterol synthesis induced by ezetimibe, the combination of ezetimibe and statins results in an incremental lowering of LDL cholesterol concentrations. The observed benefit in lipid lowering efficacy in the combined therapy is presumably predominantly caused by synergistic pharmacodynamic interactions, since the pharmacokinetics of neither statins nor ezetimibe seem to be altered in clinically relevant ways when these agents are used in combination (18). Adding 10 mg ezetimibe to a low-dose statin therapy is as effective as approximately tripling the statin dose in monotherapy, assuming an additional lipid lowering effect of 5–6 % when doubling the dose of statins (19).

Ezetimibe acts at the brush border of the small intestine and inhibits the uptake of dietary and biliary cholesterol into the enterocytes (15). It does not act via adenosine triphosphate-binding cassette (ABC)

transporters such as ABCA1, ABCG5 or ABCG8, which regulate cholesterol efflux in enterocytes (20) or via the scavenger receptor class B, type I (SR-BI), a receptor expressed in the liver and enterocytes, which is suggested to play a role in cholesterol absorption (21). Indeed, little is known about the molecular target of ezetimibe. Recently, an integral membrane protein of approximately 145 kDa could be identified in the rabbit small intestine brush border membrane as one potential component of the postulated intestinal cholesterol transporter (22). In comparison to other compounds, intestinal cholesterol absorption by ezetimibe is more pronounced than that observed for other known inhibitors of cholesterol absorption including neomycin and plant sterol and -stanol esters in humans (11). Neomycin has been shown to reduce cholesterol absorption in a dose-dependent manner by 26–49 % (23–25). Treatment with high-dose plant sterols and stanols have shown to lower cholesterol absorption by up to 45 %, but the maximal effects are observed under circumstances in which the active agents are delivered in fat carriers in conjunction with cholesterol meals (26).

The exact underlying regulatory mechanisms of the ezetimibe-induced increase in cholesterol synthesis are not known. In this context, it has been shown that plant sterols, other substances that block intestinal cholesterol absorption, increase cholesterol synthesis (27–29), decrease LDL synthesis (30), increase LDL receptor mRNA levels and LDL receptor protein concentrations but have no effect on the HMG-CoA reductase expression in peripheral mononuclear cells (29).

Mononuclear cells are a heterogeneous cell population, and, when cultured in lipoprotein-deficient serum, bind and degrade more [^{125}I]LDL than lymphocytes do (31). Furthermore, monocytes incorporate ~20-fold more [2-^{14}C]mevalonate into sterols than lymphocytes do (32). These findings suggest that cholesterol metabolism is more active in monocytes than in lymphocytes. Moreover, a positive relationship exists for LDL receptor and HMG-CoA reductase mRNA expression in mononuclear blood cells and the liver (33).

Purpose of the study

Purpose of the present study is to elucidate the mechanisms involved in the ezetimibe-induced increase in cholesterol synthesis and to examine whether changes in the LDL receptor expression contribute to its LDL-lowering effects. In specific, in the present study we will examine changes in the LDL receptor and HMG-CoA reductase mRNA concentrations under ezetimibe treatment alone (10 mg/day), under a treatment with a statin (simvastatin 40 mg/day) and under a combination treatment of ezetimibe plus simvastatin. Furthermore, since the mRNA expression provides information about effects at the transcriptional but not necessarily at the translational level, we will also analyze changes in the LDL receptor protein at the cell surface of mononuclear blood cells as well as the HMG-CoA reductase activity. As a marker of total cholesterol synthesis, the ratio of lathosterol to cholesterol in serum will be used (34), since this ratio correlates with the hepatic HMG-CoA reductase activity (35). It has been recently shown that an increased HMG-CoA reductase activity does not correlate with HMG-CoA reductase mRNA concentrations (29). This may suggest that HMG-CoA reductase activity is not only regulated at a transcriptional level, but that post-transcriptional steps are also important. In this context it has also been shown that in rats cholesterol feeding lowers HMG-CoA reductase expression at the level of translation and not at a transcriptional level (36;37). Others, however, have reported in rats a clear correlation between HMG-CoA reductase mRNA and cholesterol synthesis after cholesterol feeding (38). These contradictory findings illustrate that the mechanisms regulating HMG-CoA activity are complex and need further study.

Appendix 4:

Study protocol

Study medication

- Group A: ezetimibe 10 mg/day
- Group B: simvastatin 40 mg/day
- Group C: ezetimibe 10 mg/day plus simvastatin 40 mg/day

Study medication will be obtained free of charge through the hospital pharmacy of the University of Cologne (“Handelsware Ezetrol® 10 mg, Zocor® forte”).

There will be open label treatment.

Objectives

Primary objective

To determine the effects of ezetimibe and simvastatin on the HMG-CoA reductase activity and expression as well as on the LDL receptor expression and -protein concentration in mononuclear blood cells of healthy men.

Description of the clinical trial

Study design

A prospective, randomized, parallel 3-group study

Randomization

Eligibility for study participation will be determined by use of a standardized medical history questionnaire. Informed consent will be obtained, a physical examination and screening routine laboratory chemistry will be performed. All enrolled patients will be randomized to receive either ezetimibe, simvastatin or both.

Randomization will be performed according to a predetermined random list (balanced 6-block design) by use of sealed envelopes.

Before randomization, all selection criteria must be checked by filling in the inclusion/exclusion criteria form in the CRF.

Study Schedule

Pre-study telefone visit

- Determine and document inclusion and exclusion criteria as far as possible
- Send questionnaire

Visit 1 (qualifying visit)

- Medical history (visit to doctor's office, evaluation of standardized questionnaire)
- Informed consent
- Baseline screening (inclusion/exclusion criteria)
- Physical examination
- Baseline safety laboratory
- Fasting blood sample (study parameters V1)
- Randomization

Note: Explanation of the terms and conditions of the study to the volunteers will be executed in a seminar-like group appointment preceeding Visit 1.

Visit 2 (within 7 days after Visit 1)

- Fasting blood sample (study parameters V2)
- Dispense of medication

Visit 3 (14 days after Visit 2)

- Fasting blood sample (study parameters V3)
- End of study safety laboratory
- End of study examination
- Collection of study medication, pill count (compliance)

Study medication, dosage and duration of treatment

Medication and dosage:

- Group A: ezetimibe 10 mg/day (Ezetrol® 10 mg Tabletten), 1 tablet per day
- Group B: simvastatin 40 mg/day (Zocor® forte 40 mg) , 1 tablet per day
- Group C: ezetimibe 10 mg/day plus simvastatin 40 mg/day (Ezetrol® 10 mg Tabletten and Zocor® forte 40 mg) , 1 tablet *each* per day

The subjects will be asked to take the study medication once daily *in the evening*. There is no need to take the medication in specific relation to food consumption. The last dose should be taken approximately 12 hours before blood sampling.

Description of the method

Sample collection and handling of samples

- Fasting blood sample drawn between 7:00 and 9:00 h after a >10-h overnight fast.
- Subjects will abstain from drinking alcohol the day before and from smoking on the morning of blood sampling.
- Samples to be drawn:
  - Visit 1:
    - 1 serum monovette for routine saftety lab including lipoproteins
    - 1 EDTA monovette for blood count
    - 1 NaF monovette for plasma glucose
    - 1 serum monovette for thyroid parameters

- Visit 2:
  - 1 serum monovette for lipoproteins
  - Three 10 ml EDTA tubes for RNA analysis
  - Three 10 ml serum monovettes for other study parameters
- Visit 3:
  - 1 serum monovette for routine saftety lab including lipoproteins
  - 1 EDTA monovette for blood count
  - Three 10 ml EDTA tubes for RNA analysis
  - Three 10 ml serum monovettes for other study parameters

Handling of samples is described on separate sheet.

Study parameters (Visit 2 and 3)

- LDL receptor and HMG-CoA reductase mRNA as well as LDL receptor cell-surface protein expression will be analyzed in mononuclear blood cells.
- Serum lathosterol
- Serum lipoproteins (total cholesterol, LDL, HDL, triglycerides) → see safety parameters

Safety laboratory parameters

Visit 1
Na, K, Ca
Creatinine
GOT, GPT, γ-GT
CK
Serum lipoproteins (total cholesterol, LDL, HDL, triglycerides)
CRP
Glucose
Whole blood cell count
TSH, fT_4

Visit 2
Serum lipoproteins (total cholesterol, LDL, HDL, triglycerides)
Whole blood cell count

Visit 3
GOT, GPT, γ-GT
CK
Serum lipoproteins (total cholesterol, LDL, HDL, triglycerides)
CRP
Whole blood cell count

Number of centers and number of subjects

Number of investigational sites: This is a one-center trial.

Number of subjects: A total number of subjects of 20 in each treatment group is required (20 completed cases per group, drop-outs will be replaced). Total number: 60 completed subjects.

<u>Appendix 5:</u>

Study population

Description of the study population

Healthy men, 18–60 years of age

Inclusion criteria

- Male volunteers who are mentally and linguistically able to understand the aim of the study and to show sufficient compliance in following the study protocol
- Age between 18 and 60 years
- Patients must be generally in good or adequate health according to the judgement of the investigator and based on the history, physical examination and laboratory screening
- Body mass index between 18,5 and 30 kg/m^2
- LDL cholesterol < 190 mg/dl, triglcerides < 250 mg/dl
- Normal blood pressure (< 140/90 mmHg). Isolated systolic blood pressure (RR syst. > 140 mmHg, diast. < 90 mmHg) is acceptable if subject is otherwise in good health.
- Written informed consent before enrolment in the study

Exclusion criteria

- Intake of lipid lowering drugs within 12 weeks of study entry
- Excessive alcohol intake
- Disease of the liver (known active liver disease, e.g. chronic virus hepatitis, cholestatic liver disease, any unexplained or ongoing elevation of serum liver transaminases > 2 x ULN)
- Disease of the gastrointestinal tract (e.g. chronic inflammatory bowel disease)
- Renal dysfunction (glomerular filtration rate < 60 ml/min as calculated by the formula of Cockcroft & Gault)
- Coronary heart disease
- Eating disorders (anorexia or bulimia nervosa)
- History of recent substantial weight change, history of obesity > II° (BMI > 35 kg/m^2)
- Diabetes or other endocrine disorders
- Use of medication known to affect serum lipoprotein metabolism
- Any other disease/disorder or condition which might in the investigator’s opinion preclude the subject from participation in the study

References

1. Brown MS, Goldstein JL: A receptor mediated pathway for cholesterol homeostasis. Science 1986; 232: 34-47.

2. Brown MS, Goldstein JL: The SREBP pathway: regulation of cholesterol metabolism by proteolysis of a membrane-bound transcription factor. Cell 1997; 89: 331-340.

3. Goldstein JL, Brown MS: Regulation of the mevalonate pathway. Nature 1990; 343: 425-430.

4. Ness GC, Chambers CM: Feedback and hormonal regulation of hepatic 3-hydroxy-3-methylglutaryl coenzyme A reductase: the concept of cholesterol buffering capacity. Proc Soc Exp biol Med 2000; 224: 8-19.

5. Igel M, Sudhop T, von Bergmann K: Metabolism and drug interactions of 3-hydroxy-3-methylglutaryl coenzyme A-reductase inhibitors (statins). Eur J Clin Pharmacol 2001; 57: 357-364.

6. Sudhop T, von Bergmann K: Cholesterol absorption inhibitors for the treatment of hypercholesterolemia. Drugs 2002; 62: 2333-2347.

7. Grundy SM: Absorption and metabolism of dietary cholesterol. Annu Rev Nutr 1983; 3: 71-96.

8. Berthold HK, Sudhop T: Garlic preparations for prevention of atherosclerosis. Curr Opin Lipidol 1998; 9: 565-569.

9. Grundy SM, Mok HY: Determination of cholesterol absorption in man by intestinal perfusion. J Lipid Res 1977; 18: 263-271.

10. Law M: Plant sterol and sterol margarines and health. British Medical Journal 2000; 320: 861-864.

11. Sudhop T, Lütjohann D, Kodal A et al.: Inhibition of intestinal cholesterol absorption by ezetimibe in humans. Circulation 2002; 106: 1943-1948.

12. van Heek M, Farley C, Compton DS et al.: Ezwetimibe selectively inhibits intestinal absorption in rodents in the presence and absence of exocrine pancreatic function. Br J Pharmacol 2001; 134: 409-417.

13. van Heek M, France CF, Compton DS et al.: In vivo metabolism-based discovery of a potent cholesterol absorption inhibitor, SCH58235, in the rat and rhesus monkey through the identification of the active metabolites of SCH48461. J Pharmacol Exp Ther 1997; 283: 157-163.

14. Ezzet F, Wexler D, Statkevich P et al.: The plasma concentration and LDL-C relationship in patients receiving ezetimibe. J Clin Pharmacol 2001; 41: 943-949.

15. van Heek M, Farley C, Compton DS et al.: Comparison of the activity and disposition of the novel cholesterol absorption inhibitor, SCH58235, and its glucuronide SCH60663. Br J Pharmacol 2000; 129: 1748-1754.

16. Davis HR, Pula KK, Alton KB et al.: The synergistic hypocholesterolemic activity of the potent cholesterol absorption inhibitor, ezetimibe, in combination with 3-hydroxy-3-methylglutaryl coenzyme a reductase inhibitors in dogs. Metabolism 2001; 50: 1234-1241.

17. Zhu Y, Statkevich P, Kosoglou T et al.: Effect of ezetimibe (SCH 58235) on the activity of drug metabolising enzymes in vivo. Clin Pharmacol Ther 2000; 67: 152.

18. Kosoglou T, Meyer I, Musiol B: Pharmacodynamic interaction between the new selective cholesterol absorption inhibitor ezetimibe and simvastatin. Atherosclerosis 2000; 151: 135.

19. Jones P, Kafonek S, Laurora I, Hunninghake D: Comparative dose efficacy study of atorvastatin versus simvastatin, pravastatin, lovastatin, and fluvastatin in patients with hypercholesterolemia (the CURVES study). Am J Cardiol 1998; 81: 582-587.

20. Repa JJ, Dietschy JM, Turley SD: Inhibition of cholesterol absorption by SCH 58053 in the mouse is not mediated via changes in either the intestinal bile acid pool or the expression of mRNA for ABCA1, ABCG5 or ABCG8 in the enterocyte. J Lipid Res 2002; 43: 1864-1874.

21. Altmann SW, Davis HRJr, Yao X et al.: The identification of intestinal scavenger receptor class B, type I (SR-BI) by expression cloning and its role in cholesterol absorption. Biochim Biophys Acta 2002; 1580: 77-93.

22. Kramer W, Glombik H, Petry S et al.: Identification of binding proteins for cholesterol absorption inhibitors as components of the intestinal cholesterol transporter. FEBS Lett 2000; 487: 293-297.

23. Samuel P: Treatment of hypercholesterolemia with neomycin: a time for reappraisal. N Engl J Med 1979; 301: 595-597.

24. Kesäniemi YA, Grundy SM: Turnover of low density lipoproteins during inhibition of cholesterol absorption by neomycin. Arteriosclerosis 1984; 4: 41-48.

25. Gylling H, Miettinen TA: The effect of cholesterol absorption inhibition on low density lipoprotein cholesterol level. Atherosclerosis 1995; 117: 305-308.

26. Heinemann T, Axtmann G, von Bergmann K: Comparison of intestinal absorption of cholesterol with different plant sterols in man. Eur J Clin Invest 1993; 23: 827-831.

27. Gylling H, Puska P, Vartiainen E, Miettinen TA: Serum sterols during stanol ester feeding in a mildly hypercholesterolemic population. J Lipid Res 1999; 40: 593-600.

28. Plat J, Mensink RP: Effects of diets enriched with two different plant stanol ester mixtures on plasma ubiquinol-10 and fat-soluble antioxidant concentrations. Metabolism 2001; 50: 520-529.

29. Plat J, Mensink RP: Effects of plant stanol esters on LDL receptor protein expression and on LDL receptor and HMG-CoA reductase mRNA expression in mononuclear blood cells on healthy men and women. FASEB J 2002; 16: 258-260.

30. Gylling H, Miettinen TA: Effects of inhibitng cholesterol absorption and synthesis on cholesterol and lipoprotein metabolism in hypercholesterolemic non-insulin-dependent diabetic men. J Lipid Res 1996; 37: 1776-1785.

31. Chait A, Henze K, Mazzone T et al.: Low density lipoprotein receptor activity in freshly isolated human blood monocytes and lymphocytes. Metabolism 1982; 31: 721-727.

32. Fogelman AM, Seager J, Hokom M, Edwards PA: Separation of and cholesterol synthesis by human lymphocytes and monocytes. J Lipid Res 1979; 20: 379-388.

33. Powell EE, Kroon PA: Low density lipoprotein receptor and 3-hydroxy-3-methylglutaryl coenzyme A reductase gene expression in human mononuclear leukocytes is regulated coordinately and parallels gene expression in human liver. J Clin Invest 1994; 93: 2168-2174.

34. Kempen HJ, Glatz JF, Gevers Leuven JA et al.: Serum lathosterol concentration is an indicator of whole-body cholesterol synthesis in humans. J Lipid Res 1988; 29: 1149-1155.

35. Björkhem I, Miettinen T, Reihner E et al.: Correlation between serum levels of some cholesterol precursors and activity of HMG-coA reductase in human liver. J Lipid Res 1987; 28: 1137-1143.

36. Chambers CM, Ness GC: Dietary cholesterol regulates hepatic 3-hydroxy-3-methylglutaryl coenzyme a reductase gene expression in rats primarily at the level of translation. Arch Biochem Biophys 1998; 354: 317-322.

37. Ness GC, Keller RK, Pendleton LC: Feedback regulation of hepatic 3-hydroxy-3-methylglutaryl-coA reductase activity by dietary cholesterol is not due to altered mRNA levels. J Biol Chem 1991; 266: 14854-14857.

38. Juverics H, Hostettler J, Barrett C et al.: Diurnal and dietary-induced changes in cholesterol synthesis correlate with levels of mRNA for HMG-Coa reductase. J Lipid Res 2000; 42: 1048-1053.
